# Association between medication adherence and intrapatient variability in tacrolimus concentration among stable kidney transplant recipients

**DOI:** 10.1038/s41598-021-84868-5

**Published:** 2021-03-08

**Authors:** Hyunmin Ko, Hyo Kee Kim, Chris Chung, Ahram Han, Seung-Kee Min, Jongwon Ha, Sangil Min

**Affiliations:** 1 Department of Surgery, Seoul National University College of Medicine, Seoul, Korea; 2 Transplant Research Institute, Seoul National University College of Medicine, Seoul, Korea

**Keywords:** Medical research, Nephrology

## Abstract

This study analyzed the association between medication adherence and the intrapatient variability (IPV) of tacrolimus concentrations among kidney transplant recipients through a post hoc analysis of the dataset from a recently conducted randomized controlled trial. Among 138 patients enrolled in the original trial, 92 patients with ≥ 5 months of medication event monitoring system (MEMS) use and ≥ 4 tacrolimus trough values were included in this post hoc analysis. The variability of tacrolimus trough levels was calculated using coefficient variation (CV) and mean absolute deviation. Adherence was assessed using MEMS and self-report via the Basal Assessment of Adherence to Immunosuppressive Medication Scale. There were no statistically significant differences in the CV [median 16.5% [interquartile range 11.6–25.5%] and 16.0% [11.5–23.5%], respectively, *P* = .602] between the nonadherent (n = 59) and adherent groups (n = 33). There was also no significant correlation between the CV and adherence detected by MEMS (taking adherence, ρ = − 0.067, *P* = .527; dosing adherence, ρ = − 0.098, *P* = .352; timing adherence, ρ = − 0.113, *P* = .284). Similarly, adherence measured by self-report did not significantly affect the IPV (*P* = .452). In this post hoc analysis, nonadherent behavior, measured through electronic monitoring or self-report, did not affect the IPV.

## Introduction

Immunosuppressive (IS) agents are used for induction, maintenance, and reversal of established transplant rejection. Since the beginning of kidney transplantation, the IS regimen has continued to evolve; in particular, the use of tacrolimus has markedly increased. In 2017, the most common initial IS regimens were tacrolimus, mycophenolate mofetil (MMF), and steroids in 57.6% of recipients, followed by tacrolimus and MMF in 32.9%^1^. While the use of tacrolimus has many positive effects, it is difficult to maintain constant tacrolimus concentrations because multiple factors affect the pharmacokinetics and pharmacodynamics of tacrolimus. Key determinant factors include nonadherence, drug-drug interactions, timing and fat contents of meals, and genetic factors, such as cytochrome P450 (CYP) 3A5, CYP3A4, and adenosine triphosphate (ATP)-binding cassette sub-family B member 1 (ABCB1)^2^. Tacrolimus, therefore, is a commonly used medication with a narrow therapeutic window which is routinely monitored with therapeutic drug monitoring. Nonadherence to IS medication is known to have a large impact on graft outcome after kidney transplantation, and previous studies have reported a high rate (up to 65%) of nonadherence among kidney transplant recipients^3,4^.

Kidney transplant recipients with high intrapatient variability (IPV) of tacrolimus exposure are at higher risk of poor graft outcomes, including rejection and graft failure^5,6^. The high IPV of tacrolimus has been undoubtedly believed to be primarily the result of nonadherent behavior. It is also sometimes used as a surrogate marker for nonadherence in clinical studies^7^; a recent study suggested that high IPV can be considered a proxy measure of nonadherence^8^. However, it remains unclear how important nonadherence is in determining IPV or whether calculated IPV based on tacrolimus levels measured during outpatient visits reflects nonadherent behavior. This poor understanding partly results from the multifactorial nature of medication nonadherence and from the inherent limitations of clinical methods used to measure medication nonadherence. Nonadherence is defined as deviation from the prescribed medication regimen sufficient to adversely influence the regimen’s intended effect^9^. Clinical methods to measure adherence, such as pill counts, questionnaires, and patients’ diaries, can over- or underestimate adherence^10^. Instead, it has become widely accepted that electronic monitoring (EM) provides the best estimate of adherence^11^. The Food and Drug Administration endorsed “smart bottles” as a tool to improve drug development trials by confirming protocol fidelity. EM is an objective method of detecting nonadherence in nature and has been proven useful in quantifying medication adherence and associated clinical outcomes in prospective studies involving adult kidney transplant recipients^12^. In this context, data from clinical trials using EM of tacrolimus may provide the best opportunity to investigate the association between tacrolimus IPV and nonadherence.

Therefore, the aim of this study was to analyze the association between medication adherence and the IPV of tacrolimus concentrations among kidney transplant recipients, using the dataset from a recently conducted randomized controlled trial (RCT).

## Results

### Patient’s characteristics

A total of 92 kidney recipients were eligible for this post hoc analysis. The median values of CV and MAD for the total population were 16.4% (IQR 11.3–24.9%) and 13.1% (IQR 8.5–19.2%), respectively. The median age at transplant was 43 years (IQR 18–67), and the median post-transplant period was 1.8 years (IQR 1.1–3.7). There were 58 males (63.8%), and the mean body mass index was 22.0 ± 3.1 kg/m^2^. Thirty-seven (40.2%) recipients received kidneys from deceased donors, and 55 (59.8%) received kidneys from living donors. Regarding medication regimens, 86 (93.5%) patients received MMF, and 86 (93.5%) patients received prednisone. Using MEMS data, the nonadherent group was defined as < 98% taking adherence and/or at least one drug holiday, as described previously^13^. There was no difference in baseline characteristics between the adherent and nonadherent groups (Table 1). Taking adherence measured by MEMS was 99.7% (99.2–100.0), 86.0% (63.9–95.9), and 96.3% (77.7–99.5) in the adherent, nonadherent, and total study groups, respectively (*P* < 0.001).

**Table 1:** Patient demographics.

	Total (n = 92)	Adherent (n = 33)	Non-adherent (n = 59)	*P*
Age at transplant, year, median (IQR)	43 (18–67)	43 (21–67)	43 (18–58)	.078
Length of time post-transplant, year (IQR)	1.8 (1.1–3.7)	1.7 (1.1–3.2)	1.8 (1.1–3.9)	.663
Sex, male (%)	58 (63.0)	22 (66.7)	36 (61.0)	.590
BMI, mean (SD), kg/m^2^	22.0 (3.1)	21.9 (3.3)	22.1 (3.0)	.801
**Education (%)**				.828
Undergraduate college	46 (50.0)	16 (48.5)	30 (50.8)	
Graduate college	46 (50.0)	17 (51.5)	29 (49.2)	
**Donor type (%)**				.904
Deceased	37 (40.2)	13 (39.4)	24 (40.7)	
Living	55 (59.8)	20 (60.6)	35 (59.3)	
**Immunosuppressive (%)**				
MMF	86 (93.5)	30 (90.9)	56 (94.9)	.455
Prednisone	86 (93.5)	31 (93.9)	55 (93.2)	.893
**Daily tacrolimus dose, mean ± SD, mg**				
At enrollment	4.5 ± 2.4	4.1 ± 1.7	4.7 ± 2.6	.229
At last visit	4.1 ± 2.1	3.9 ± 1.7	4.3 ± 2.3	.292
**Trough concentration (C0) of tacrolimus, mean ± SD, ng/mL**				
At enrollment	5.8 ± 1.8	6.4 ± 2.2	5.4 ± 1.5	.008
At last visit	5.8 ± 1.6	6.0 ± 1.5	5.7 ± 1.7	.302
**MEMS, median (IQR), %**				
Taking adherence	96.3 (77.7 – 99.5)	99.7 (99.2 – 100.0)	86.0 (63.9 – 95.9)	< .001
Dosing adherence	89.7 (65.2 – 97.0)	97.8 (95.9 – 99.5)	72.5 (53.3 – 89.8)	< .001
Timing adherence	90.9 (61.6 – 98.0)	98.1 (96.5 – 99.3)	73.5 (50.0 – 90.1)	< .001
TTR, median (IQR)	47.7 (17.5 – 76.5)	50.1 (17.5 – 82.3)	43.1 (14.9 – 71.0)	.552

IQR, interquartile range; BMI, body mass index; SD, standard deviation; MMF, mycophenolate mofetil; MEMS, medication event monitoring system; TTR, time in therapeutic range.

### Differences in IPV indices between adherent and nonadherent groups

**Figure 1:**
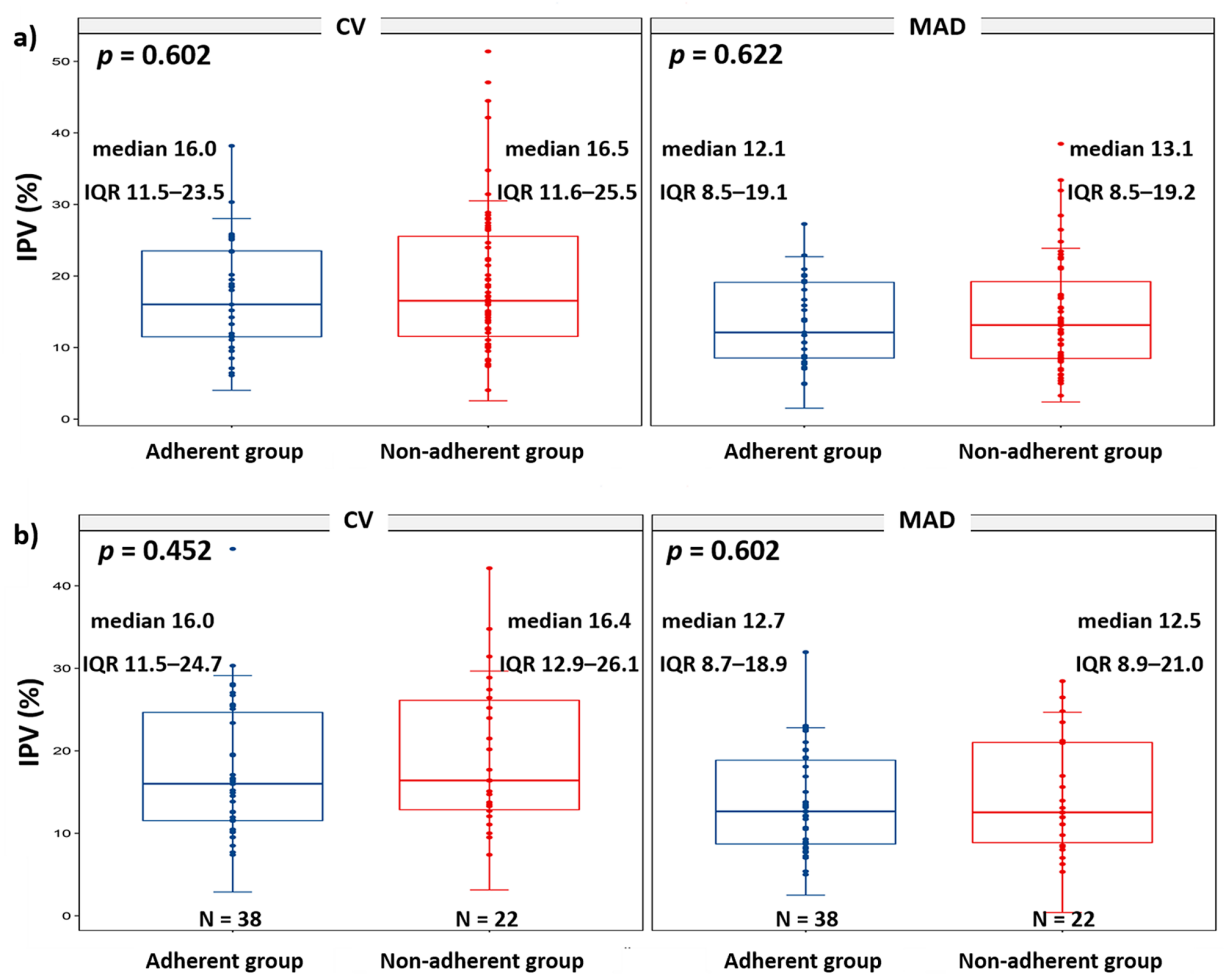
Differences in IPV indices between adherent and nonadherent groups measured through an electronic monitoring system (**a**) and self-report (**b**). CV, coefficient variation; IPV, intrapatient variability; IQR, interquartile range; MAD, mean absolute deviation.

There were no statistically significant differences in either the CV (median 16.5 [IQR 11.6–5.5] and 16.0 [11.5 23.5], respectively, *P* = 0.602) or the MAD (median 13.1 [IQR 8.5–19.2) and 12.1 [8.5–19.1], respectively, *P* = 0.622] between the nonadherent group (n = 59) and the adherent group (n = 33) (Fig. 1a). Furthermore, the IPV was not different between groups defined by various cutoff values of taking adherence (Supplementary Table S1).

The median value of TTR in the total population was 47.7% (IQR 17.5–76.5). Like CV and MAD, there were no statistically significant differences in the TTR (median 50.1 [IQR 17.5–82.3] and 43.1 [14.9–71.0], respectively, *P* = 0.552) between the two groups.

In adherence measured through self-report, over time, adherent patients gradually decreased (28 days, 62; 90 days, 49; 180 days, 46), and non-adherent patients gradually increased (28 days, 30; 90 days, 43; 180 days, 46). Three self-report checks during the 6-month follow-up revealed that 38 patients were all adherent, and 22 patients were all non-adherent. There were no significant differences in the IPV indices between these two groups (CV, *P* = 0.452; MAD, *P* = 0.602) (Fig. 1b).

### Correlation between IPV indices and adherence measured by MEMS

**Figure 2:**
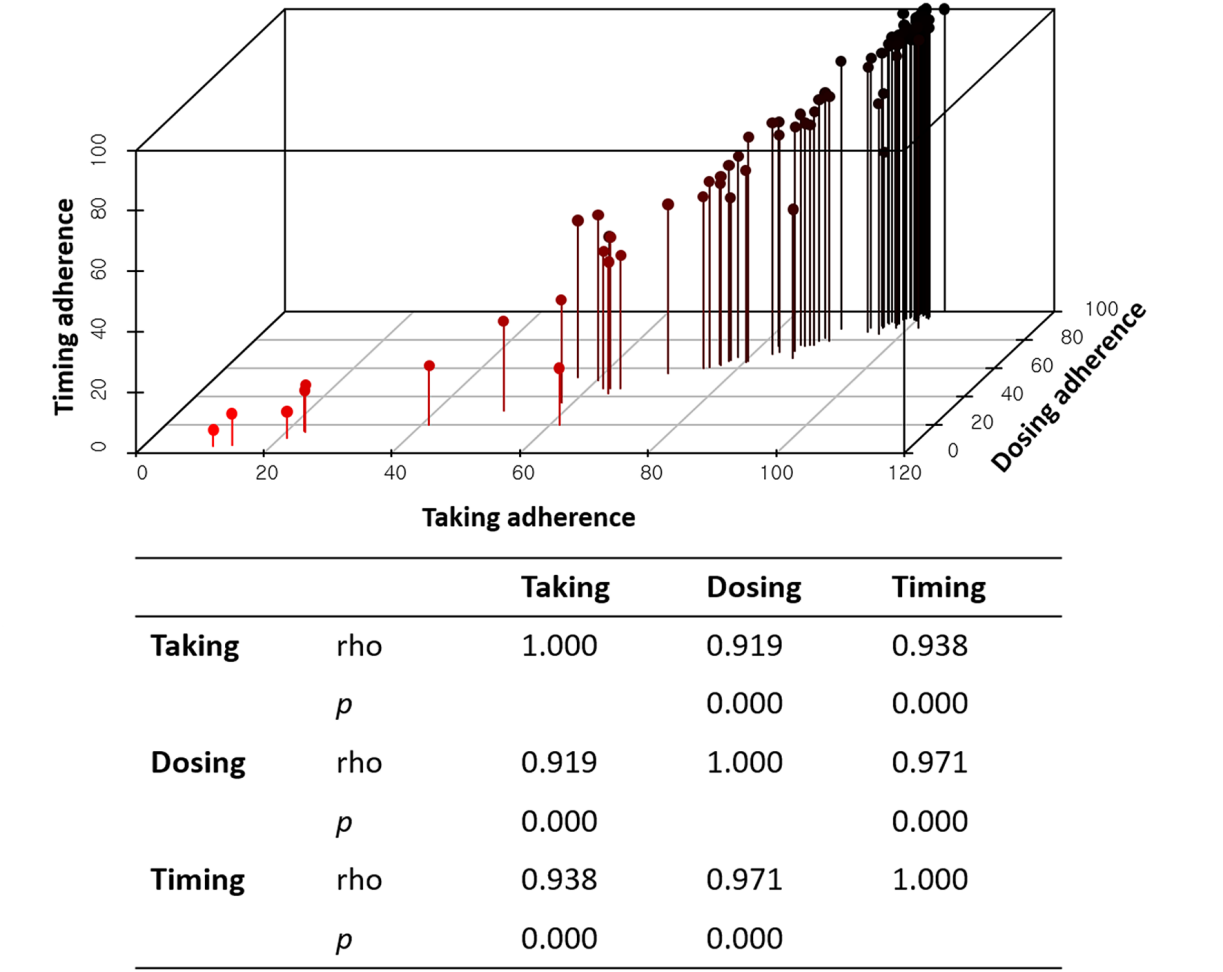
Correlation between intrapatient variability indices and adherence measured by the medication event monitoring system.

We analyzed the correlation between taking, dosing, and timing adherence. There was a linear correlation among the three parameters of MEMS (taking vs. dosing, rho = 0.919, *P* < 0.001; taking vs. timing, rho = 0.938, *P* < 0.001; dosing vs. timing, rho = 0.971, *P* < 0.001) (Fig. 2).

**Figure 3:**
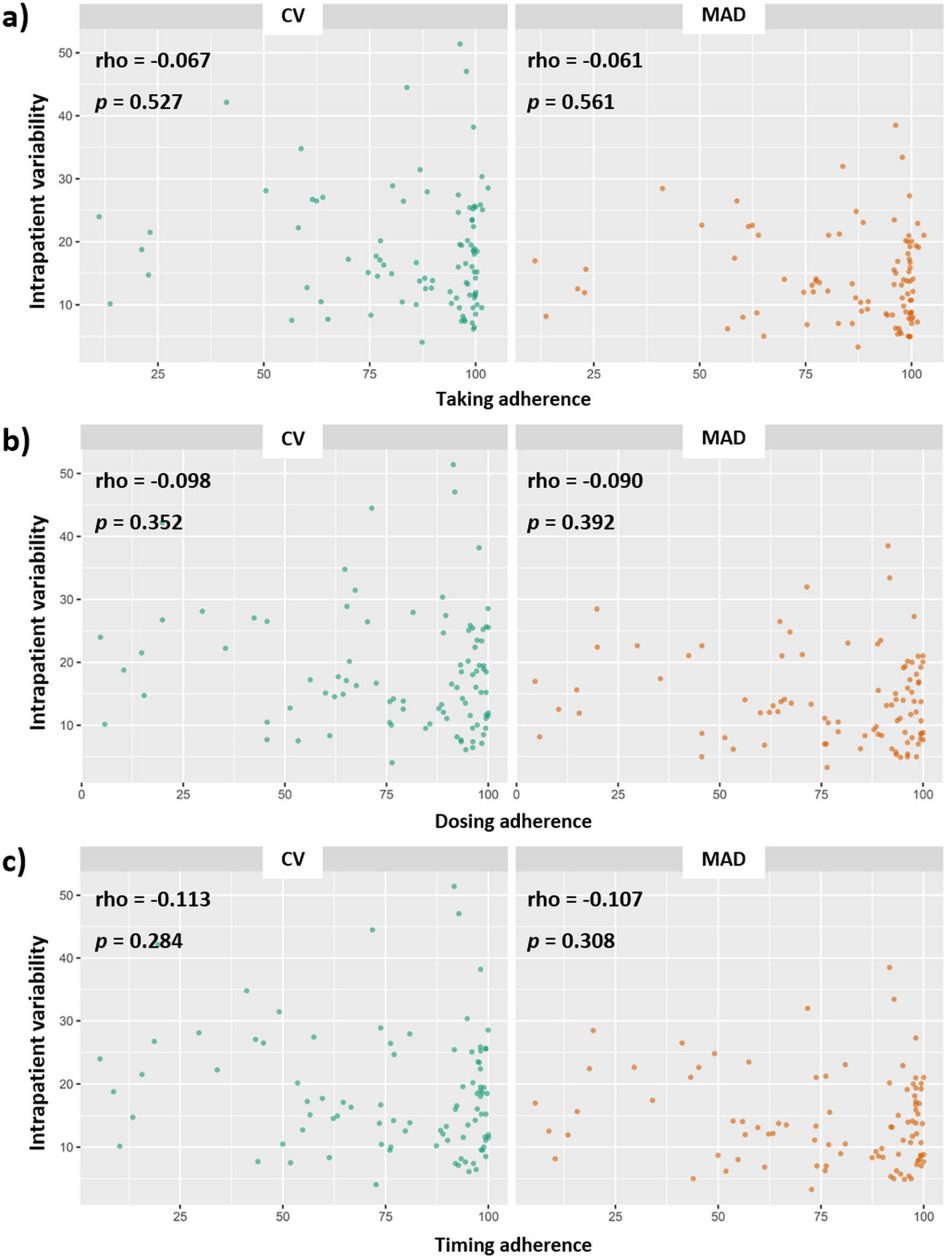
Correlation analysis using Spearman’s rho to identify correlations between adherence measures using the medication event monitoring system and intrapatient variability indices. Taking adherence (**a**), dosing adherence (**b**), timing adherence (**c**). CV, coefficient variation; MAD, mean absolute deviation.

Correlation analysis using Spearman’s rho failed to find a significant correlation between longitudinal adherence measured by MEMS (taking adherence, ρ = − 0.067, *P* = 0.527; dosing adherence, ρ = − 0.098, *P* = 0.352; timing adherence, ρ = − 0.113, *P* = 0.284) and IPV indices. Figure 3 shows the results as a scatter plot.

### Clinical outcomes

**Figure 4:**
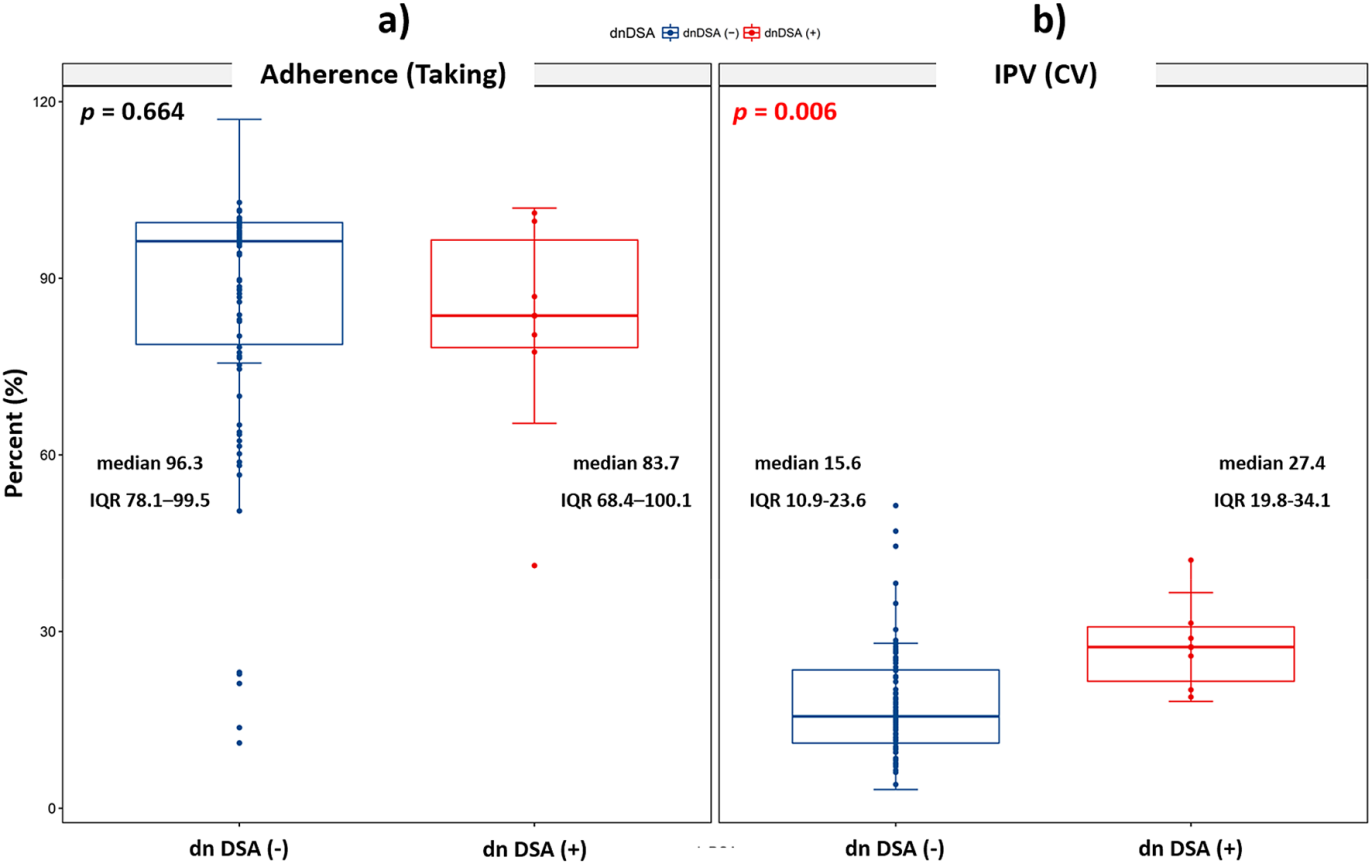
The development of dnDSA in terms of taking adherence (**a**) and IPV (**b**). IPV, intrapatient variability; CV, coefficient variation; dnDSA, de novo donor-specific antibodies; IQR, interquartile range.

The development of dnDSA was found in six patients (two in the adherent group and four in the nonadherent group; *P* = 0.893). Taking adherence was not significantly different between the dnDSA-negative (median 96.3 [IQR 78.1–99.5]) and -positive groups (median 83.7 [IQR 68.4–100.1]) (*P* = 0.664) (Fig. 4a). However, the CV was significantly higher in the dnDSA-positive group (CV 27.4, IQR 19.8–34.1) compared to the dnDSA negative group CV 15.6, IQR 10.9–23.6) (*P* = 0.006) (Fig. 4b). Changes in the eGFR using the Modification of Diet in Renal Disease Study also did not differ between the two groups at 1, 3, and 5 years after enrollment in the study (Supplementary Table S2).

## Discussion

In this study, we performed post hoc analysis to investigate the relationship between adherence and tacrolimus IPV using the dataset from the original RCT. As expected, high IPV was associated with dnDSA development, which was concordant with previous reports^5,6^. It has been assumed that nonadherent behavior may be related to high IPV; however, no literature had provided clear evidence until now. We used EM and defined nonadherence as < 98% taking adherence and/or at least one drug holiday, based on a previous study^14^. We also found no clear relationship between nonadherence and tacrolimus IPV in stable renal transplant patients. This finding is completely concordant with a report from the Leiden group^15^. We also attempted to compare various cutoff values (taking adherence 95%, 90%, 80%, 70%, and 50%) because we had concerns that the cutoff value of nonadherence was too high; however, we did not find any significant relationship. A recent study using dried blood spot technology also failed to show a clear relationship between nonadherence and tacrolimus IPV; they recommended that the correlations must be more closely revealed before IPV can be used as a surrogate marker of nonadherence^16^.

As reported in previous studies, tacrolimus IPV was significantly higher in the dnDSA-positive group in this study^17,18^. On the contrary, we could not find any association between adherence and dnDSA development in stable renal transplant recipients. Therefore, we need an alternate explanation for tacrolimus IPV instead of nonadherence.

Trough level collection time could be regarded as a cause of high IPV in outpatient clinics. However, high IPV caused by differences in blood collection time may not adversely affect graft outcome. The timing and fat contents of meals affect the likelihood of high IPV. Tacrolimus is absorbed by the small intestine, and bioavailability is about 25%^4^. Kimikawa et al. compared trough concentrations between preprandial and postprandial oral administration and found trough concentrations to be effectively absorbed by preprandial oral administration^19^. In addition, a high-fat meal reduces the rate of tacrolimus absorption relative to a low-fat meal^20^. Therefore, we always recommend that patients take tacrolimus on a gastric empty status (2 h after meals and 1 h before meals) based on hospital protocol. Drug-drug interaction (for example, proton pump inhibitors and calcium channel blockers) is also a key factor in increased tacrolimus IPV. Therefore, kidney recipients need to be careful when taking other medications^21^. Genetic factors may also affect the development of high IPV. Stifft et al. reported a decrease in IPV when converting tacrolimus to once-daily in stable renal transplant patients, especially in patients with the CYP3A5*1/3 genotypes^22^. Other studies have reported that CYP3A5 polymorphism affects the achievement of target tacrolimus trough levels and increases early acute rejection^23^.

Immunosuppressive medications are known to have a critical effect on outcome after kidney transplantation. Thus, the nonadherent behavior of recipients to IS has long been a concern for transplant specialists. Adherence to medication is generally defined as the extent to which patients take medications as prescribed by their health care providers^24^. Early declining medication nonadherence is associated with chronic nonadherence and eventually leads to adverse clinical outcomes^25^. Contrary to expectations, using a mobile medication management application did not contribute to the improvement of adherence rate (overall nonadherence rate: mobile group, 65% and control group, 62.1%, respectively; odds ratio, 1.14; *P* = 0.89). This was attributed to the early discontinuation of the mobile application and the failure to provide strategies to facilitate patient engagement with the application.

There were several limitations to our study. A 6-month period of follow-up for adherence may not sufficiently represent the patients’ adherence behavior. However, adherence usually peaks in the early period after transplantation and declines thereafter. Thus, a measured 6-month period of adherence 1 year post-transplant was a reasonable approach to investigate the possibility of a relationship between adherence and IPV and its effects on the long-term transplant outcome. In addition, our study population had a low IPV overall. In previous studies, a criterion affecting clinical outcome was presented with a CV value of 30^16^. In this study, only eight out of 92 had a CV > 30. The study population consisted of motivated patients from an RCT and was composed of a single ethnic group. Therefore, the results cannot be extrapolated to patients with different ethnicities in other regions. Lastly, the differences between previously described factors affecting IPV were not compared. Our patients used a twice-daily formulation of tacrolimus. A once-daily formulation could have increased adherence and been associated with low IPV. Therefore, further studies are required to define the impact of once-daily formulations on adherence and IPV.

In conclusion, we were unable to determine a clear relationship between nonadherent behaviors measured through EM or self-report and tacrolimus IPV in stable renal transplant patients. This result is contrary to what has been considered and implies a lack of clear understanding of the mechanism of tacrolimus IPV which may impact long-term transplant outcomes. More advanced study designs are needed to clarify this.

## Materials and methods

This study was conducted using the dataset from a previously reported PRIMA (imPRoving adherence to Immunosuppressive therapy by Mobile internet Application) trial^26^. The PRIMA trial was a prospective RCT evaluating the effectiveness of 6 months of use of the medication manager application in promoting medication adherence in patients at more than 1 year post-transplant (IRB no. 1306-031-496, Clinicaltrials.gov: NCT 01905514). This post hoc analysis was conducted according to the Declaration of Helsinki and approved by the Seoul National University Hospital Institutional Review Board (IRB No. 2007-059-1140). The written informed consent was obtained from each patient, and also from parents and/or legal guardians for minor age group participants.

### Study population

The inclusion criteria of the original RCT were patients aged 15 to 70 years on twice-daily tacrolimus (PROGRAF; Astellas Pharma, Tokyo, Japan), who were over 1 year post renal transplantation. Multi-organ transplantation and pregnant recipients were excluded. Of the total 138 patients enrolled in the original RCT, we included patients with the use of more than 5 months of EM and with four or more measured outpatient tacrolimus level values in this post hoc analysis.

The primary outcome of the analysis was the comparison of the IPV and the time in therapeutic range (TTR) between adherent and nonadherent groups. In addition, we reviewed de novo donor-specific antibodies (dnDSA) incidence and estimated glomerular filtration rate (eGFR) changes between the groups.

### Adherence measures

Adherence was measured using EM and self-reports. Three parameters (taking, dosing, and timing) were measured using the Medication Event Monitoring System (MEMS)-V TrackCap EM system (Aardex, Ltd., Zug, Switzerland) for 6 months after enrollment. Taking adherence was defined as the percentage of cap removals compared with the number prescribed for the monitoring period. Dosing adherence was defined as the percentage of days that the patient took the prescribed number of doses. Timing adherence was defined as the percentage of correct dosing intervals plus/minus 1 h of prescribed intake timing. MEMS lids recorded the time and date of bottle opening onto a digital chip, and individual data were downloaded by the study pharmacists using a MEMS reader software program during the participants’ scheduled visit. The cutoff of the nonadherent group was < 98% taking adherence and/or at least one drug holiday based on previous studies^13^.

Self-reported adherence was measured using the Basel Assessment of Adherence to Immunosuppressive Medication Scale (BAASIS) based on a 4-week recall on days 28, 90, and 180. The BAASIS is a questionnaire of four items: taking and timing of medication use, drug holidays, and dose reduction. Patients with a score of 1 or more in any items of the BAASIS were considered nonadherent^27^.

### Tacrolimus variability

The concentration of tacrolimus was measured by high-performance liquid chromatography-tandem mass spectroscopy with a Waters 2795 Alliance HT system (Micromass, Manchester, UK) from whole blood samples drawn just prior to taking the morning dose in outpatient basis^28^. The accuracy was from 96.0 to 104.0%. The intraday coefficient variation (CV) varied from 5.2 to 9.3%, and the interday CV ranged from 3.6 to 9.6%. The lower limit of quantification for tacrolimus was 0.8 ng/mL.

Tacrolimus doses were adjusted at the discretion of the attending physician during the original study period in order to maintain its’ target concentration between 4 and 6 in recipients. We used tacrolimus concentrations for calculating tacrolimus IPV instead of dose-normalized concentrations. Because dose normalization is a measure of variability of tacrolimus absorption and an indicator for clearance rather than exposure, dose normalization was not used when evaluating tacrolimus IPV^8^.

The tacrolimus IPV was calculated using formulas based on previous literature^29^:

$$ Coefficient\;of\;variation\,\,\left( {CV,\% } \right) = SD/X_{mean} \times 100; $$

*Mean absolute deviation (MAD, %)* = ([|*X*_*mean*_ *−* *X*_*1*_| +|*X*_*mean*_ *−* *X*_*2*_|… +|*X*_*mean*_ *−* *X*_*n*_|]/n)/*X*_*mean*_ × *100*, where *SD* is the standard deviation of the tacrolimus concentrations, and *X*_*mean*_ is the mean tacrolimus concentration during the period of adherence measurement.

The TTR was calculated using the Rosendaal method, estimating the number of days within range^30^.

### Statistical analysis

The data are expressed as median ± interquartile range (IQR) or mean ± SD. Comparisons of CV and MAD between groups were analyzed using an independent t test. A correlation analysis (Spearman’s rho) was performed to evaluate the association between IPV and longitudinal adherence or among adherence parameters measured by MEMS.

A two-sided *P*-value < 0.05 was considered statistically significant. Statistical analyses were performed using SPSS (version 21.0 for Windows; IBM Corp., Armonk, NY).

## Supplementary Information

Supplementary Information

## Abbreviations

BAASIS: Basal Assessment of Adherence to Immunosuppressive Medication Scale
CV: Coefficient variation
CYP: Cytochrome P450
dnDSA: De novo donor-specific antibodies
eGFR: Estimated glomerular filtration rate
EM: Electronic monitoring
IS: Immunosuppressive
IPV: Intrapatient variability
IQR: Interquartile range
MAD: Mean absolute deviation
MEMS: Medication event monitoring system
MMF: Mycophenolate mofetil
PRIMA: ImPRoving adherence to Immunosuppressive therapy by Mobile internet Application
RCT: Randomized controlled trial
TTR: Time in therapeutic range
