# Supplementary material for: Association between medication adherence and intrapatient variability in tacrolimus concentration among stable kidney transplant recipients
Source: Sci Rep. 2021 Mar 8;11:5397. doi: 10.1038/s41598-021-84868-5 (PMC7940492; doi:10.1038/s41598-021-84868-5)
Supplement: Supplementary file 1 — Supplementary Information [file 41598_2021_84868_MOESM1_ESM.docx]

**Supplementary Table S1.** Comparison of groups distributed by various cutoff values of taking adherence

| Cutoff Taking adherence | CV (C0) Median [IQR] | | *P-*value |
| --- | --- | --- | --- |
|  | **Adherent** | **Non-adherent** |  |
| 95% | 16.54 [11.07–24.67] | 16.31 [12.32–26.46] | 0.629 |
| 90% | 16.04 [10.64–24.09] | 16.68 [12.65–26.49] | 0.396 |
| 80% | 15.60 [11.07–24.98] | 17.47 [13.18–25.86] | 0.379 |
| 70% | 16.01 [11.19–23.48] | 21.86 [11.04–26.97] | 0.201 |
| 50% | 16.17 [11.08–25.12] | 20.13 [13.58–28.52] | 0.393 |

CV, coefficient variation; IQR, interquartile range

**Supplementary Table S2.** Changes in eGFR at 1, 3, and 5 years after enrollment in study

|  | **1 year** Mean ± sd | **3 year** Mean ± sd | **5 year** Mean ± sd |
| --- | --- | --- | --- |
| Total | -0.19 ± 7.04 | 0.37 ± 9.77 | -0.94 ± 14.22 |
| Adherent | -0.42 ± 7.10 | 0.37 ± 7.83 | -0.22 ± 12.38 |
| Non-adherent | -0.05 ± 7.06 | 0.37 ± 10.77 | -1.34 ± 15.24 |
| *P*-value | 0.811 | 0.997 | 0.720 |
| Low IPV  (>CV median 16.4) | -0.42 ± 5.91 | 0.52 ± 9.72 | -0.63 ± 13.92 |
| High IPV  (<CV median 16.4) | -0.05 ± 8.07 | 0.22 ± 9.93 | -1.24 ± 14.63 |
| *P-*value | 0.755 | 0.882 | 0.837 |

eGFR, estimated glomerular filtration rate, sd, standard deviation; IPV, intrapatient variability; CV, coefficient variation
